# Confidence and Receptivity for COVID-19 Vaccines: A Rapid Systematic Review

**DOI:** 10.3390/vaccines9010016

**Published:** 2020-12-30

**Authors:** Cheryl Lin, Pikuei Tu, Leslie M. Beitsch

**Affiliations:** 1 Policy and Organizational Management Program, Duke University, 2204 Erwin Rd, Durham, NC 27705, USA; c.lin@duke.edu; 2 Department of Behavioral Sciences and Social Medicine, Florida State University College of Medicine, 1115 W. Call St, Tallahassee, FL 32306, USA; les.beitsch@med.fsu.edu

**Keywords:** vaccines, vaccine hesitancy, immunization, public health, health behavior, public opinion, communication, infectious diseases, pandemic, coronavirus

## Abstract

While COVID-19 continues raging worldwide, effective vaccines are highly anticipated. However, vaccine hesitancy is widespread. Survey results on uptake intentions vary and continue to change. This review compared trends and synthesized findings in vaccination receptivity over time across US and international polls, assessing survey design influences and evaluating context to inform policies and practices. Data sources included academic literature (PubMed, Embase, and PsycINFO following PRISMA guidelines), news and official reports published by 20 October 2020. Two researchers independently screened potential peer-reviewed articles and syndicated polls for eligibility; 126 studies and surveys were selected. Declining vaccine acceptance (from >70% in March to <50% in October) with demographic, socioeconomic, and partisan divides was observed. Perceived risk, concerns over vaccine safety and effectiveness, doctors’ recommendations, and inoculation history were common factors. Impacts of regional infection rates, gender, and personal COVID-19 experience were inconclusive. Unique COVID-19 factors included political party orientation, doubts toward expedited development/approval process, and perceived political interference. Many receptive participants preferred to wait until others have taken the vaccine; mandates could increase resistance. Survey wording and answer options showed influence on responses. To achieve herd immunity, communication campaigns are immediately needed, focusing on transparency and restoring trust in health authorities.

## 1. Introduction

The COVID-19 pandemic persists with resurgent waves while debates intensify about reinstituting lockdowns, civil liberties, and societal livelihood. Vaccines have become the hopeful savior to end the worst global health and economic crisis of living memory. Beyond the complex logistics of developing and testing, mass manufacturing, and distribution, the public’s confidence and acceptance for the vaccines are unclear and changing [1,2], rendering achieving herd immunity challenging.

Vaccine hesitancy can be dated back to the 1800s [3]. Well before the pandemic, the World Health Organization in 2019 identified it as a top global health threat [4]. Studies regarding intention to get vaccinated against COVID-19 have been published since early 2020 with great variations in question formats and results [5,6,7,8,9,10]. Many reported a pattern of increasing doubts about vaccine safety and declining receptivity [11,12,13]. However, differences in their findings and factors associated with vaccine hesitancy unique to COVID-19 have not been systematically examined.

A comprehensive understanding of the current vaccine sentiment and potential determinants of people’s behavior is critical for planning effective health communications to encourage uptake and successfully implementing population immunization. The objectives of this rapid review are to (a) compare the trends of the public’s reception and rejection of COVID-19 vaccines over time across national and international polls; (b) assess the impact of survey design, particularly the wording of the questions and framing of the answer choices, on responses; and (c) analyze factors pertaining to vaccine perceptions, concerns, and intention during the pandemic.

## 2. Materials and Methods

The research questions for this review are: (1) how have confidence and receptiveness for COVID-19 vaccines changed; (2) does survey design affect responses; and (3) what factors are associated with vaccination decision, unique to COVID-19. To help inform policy makers, health departments, and healthcare professionals in a timely manner, we streamlined the systematic review process for a rapid review [14]. The attenuated process included limiting searches to publications in English and not posting protocol to a systematic review registry.

### 2.1. Data Sources and Searches

Two searches were performed to identify studies published between 1 January and 20 October 2020 surveying people’s willingness to get a COVID-19 vaccine. The first literature search followed the systematic review procedure on PubMed, Embase, and PsycInfo with search terms: (COVID-19 OR coronavirus OR SARS-CoV-2) AND (vaccine OR immunization) AND (survey OR questionnaire OR poll). The search strategy included MeSH terms and free-text word variations adjusted for each database (details in Appendix A Table A1, Table A2 and Table A3).

The second search was conducted on Google using iterating combinations of key words including “COVID-19,” “coronavirus,” “vaccine,” “survey,” “poll,” “hesitancy,” and “willingness.” COVID-19-related news, web posts, and polls were scanned for the survey item of interest. Auxiliary key words (e.g., Gallup, The Economist) were used to locate affiliated questionnaires in series. When a potential survey was cited in an article, the original press release or official report was sought. If a report provided insufficient information, the respective organization was contacted.

### 2.2. Study Selection

Peer-reviewed studies were selected according to the Preferred Reporting Items for Systematic Reviews and Meta-Analyses (PRISMA) guidelines from the three databases [15]. The inclusion criteria were primary research that included at least one survey question on willingness, confidence, or intention of getting a COVID-19 vaccine if/when available, conducted in any country, and published in English. Titles and abstracts of the search results were screened, followed by full-text reviews by two researchers to determine eligibility; disagreements were resolved through consensus.

News articles and official reports (preferred, if available) from online searches and organization websites were included if they presented the question and responses to the survey item of interest. Surveys not explicitly asking about the receptiveness of COVID-19 vaccines or only discussing reasons for objecting vaccinations were excluded.

### 2.3. Data Extraction, Analysis, and Quality Assessment

Two researchers independently extracted data, verified by another researcher. The details summarized included surveying dates and authors or organizations, whether it was a one-time or longitudinal study, sampling size and method, key question(s), answer choices and responses, relevant factors and their effects. Findings were synthesized narratively and presented both in a summary table and graphs to illustrate the trends over time. The wording of questions and answer options were analyzed for response differences across surveys. To facilitate a rapid review and address limitations posed by the observational nature of surveys, study quality was assessed by survey administration, sampling method, size, and representativeness in lieu of utilizing a formal measure. Small, informal surveys were excluded.

## 3. Results

### 3.1. Search Results and Survey Characteristics

**Figure 1:**
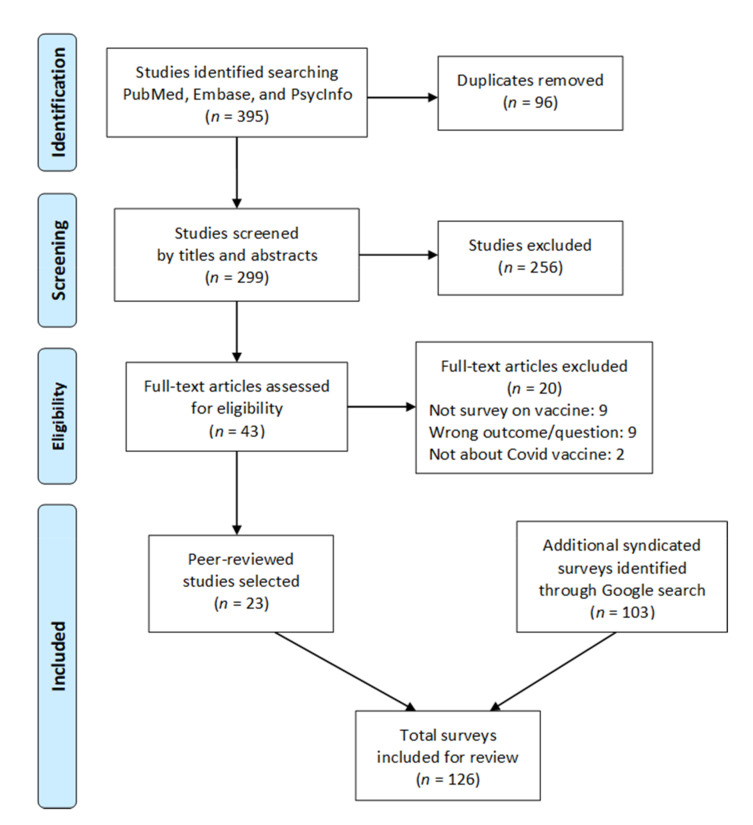
PRISMA flow diagram of study search and selection.

A total of 126 surveys was selected for this review, including 23 academic studies that passed the multi-level screening originating from 299 results from PubMed, Embase, and PsycInfo searches (Figure 1). All included surveys were conducted as either self-reported online questionnaires or phone interviews.

Overall, the research design and data collection procedures of the included studies were deemed appropriate, conducted mostly by reputable pollsters (e.g., Ipsos, Pew Research, USA Today). Common quality issues included unreported non-response rates and not explicitly describing whether the percentage tabulations included missing data. A large majority of the surveys polled 1000–3000 participants and five polled over 10,000. All except seven had national/state representative samples through random selection from the targeted population (e.g., via voter registration or phone numbers) or large national/international opt-in panels, many stratified by demographics. The other seven were convenience samples utilizing social media [2,7,16,17,18] or recruiting posters [19,20].

The majority of the surveys were US-based; 16 (12.7%) international surveys covered a total of 31 countries, predominately conducted in the earlier study period. Eighty-seven of the surveys were among 15 series of recurring polls. Two major longitudinal surveys were conducted by Morning Consult with 33 surveys starting late February and YouGov (partnering with The Economist and Yahoo News) with 17 surveys since May. The summary table in Appendix A Table A4 described each survey’s dates, country (if non-US), sample size, question wording, answer options, responses, key findings, and relevant factors.

### 3.2. Trends in Vaccine Acceptance, Hesitancy, and Refusal

There have been substantial variations in COVID-19 vaccine receptivity between countries [8,20,21,22], states within the US [9,23,24,25,26], and subgroups. Among America surveys, the highest intended acceptance of 72% was reported by Morning Consult in early April [27]. In mid-October, the propensity dropped to its lowest at 48% (men 55%, women 42%) [11]. Regionally, acceptance ranged from 38% in the Northeast to 49% in the West [28]. Internationally, some Asian countries had higher acceptance: 88.6–91.3% in China and 79.8% in South Korea—both also reported higher trust in central governments [21,29]. Other highly receptive countries included Brazil (85.36%), South Africa (81.58%), Denmark (80%), and the UK (79%); Russia had the lowest (54.9%), followed by France (58.9–62%), which also reported the largest rate of “unsure” responses (28%) [21,22].

#### 3.2.1. Demographics Variables

Demographic characteristics were common subgroup variables cross-tabulated with vaccination intention. Of note is the growing gap between those without and with college degrees [5,10,24,30]; one survey recorded 42% and 62%, respectively, with 73% for post-graduates [31]. Individuals with lower income [24,32,33,34], uninsured [2,32], living in rural areas [1,35,36] or larger households [1] were less likely to get vaccinated.

People over 55 or 65 (depending on each survey’s categorization) remain the most receptive among age groups [21,22,30,36,37,38], often followed by the youngest, 18–24 or –34 groups [6,16,24,39,40], while other polls found younger age had lower acceptance [1,31,41]. A majority of the US-based surveys reported lower intentions among women than men [1,24,30,37,42], while some international polls found the opposite [10,21,34]. A multi-country study reported that women’s vaccine refusal was more than double men’s [22].

White Americans consistently expressed higher receptivity [33,35] and Blacks showed more suspicion and lower confidence in the vaccine [2,30,32,33,43]. One study found Blacks were 40% more likely than Whites to reject due to lack of trust in vaccine safety, efficacy, and resources [42]. Divergent results were reported among Hispanics—some reported higher [1,24,33,39,44,45] or similar [30] acceptance as Whites, though others found lower [31,46]. Asian Americans were included in a few surveys for subgroup analysis, and they expressed greater acceptance [24,30,47] (e.g., 81% of Asian Americans vs. 68% of Whites and Hispanics, 40% of Blacks [9]).

#### 3.2.2. Vaccine Attributes and Individual Factors

Some surveys queried factors relevant to vaccination decisions. Vaccine attributes posed prevalent concerns, particularly due to the newness of COVID-19 vaccines [22,48]. The most commonly cited reasons for hesitation or refusal were fear of side effects [1,22,47,49,50], safety [5,34,37,51], and effectiveness [2,5,24,52,53,54]. Belief that vaccines are unnecessary [43,50,52,55], inadequate information [34,47,50], unknown/short duration of immunity [2,5,50,53], and a general anti-vaccine stand [1,10,55] were associated with lower acceptance.

Less frequently discussed were cost [47], willingness to pay [2,34,56], and country of vaccine origin. Cost ranked low as a concern for Americans [5]. One survey reported that 17.1% would get the vaccine only if covered by insurance [54] and another found 49% expected it to be free (paid by insurance or government) [56]. In America, US-made vaccines were more trusted than China-made [53,57] or foreign-developed [5,53]. Most Chinese (64%) expressed no preference for domestic or foreign-made [34].

National (but not necessarily state) coronavirus infection and mortality rates [9,21,23,58], perceived risk of infection [2,7,42] and disease severity [2,53] were predictors of vaccination intentions. The impacts of having been infected oneself or knowing a friend/family who had and the desire to protect oneself or others were also cited but less conclusive. Some studies indicated positive association [19,53], while others found no correlation [21]. One study reported only 55% of those worrying about themselves or family members getting infected would get vaccinated [49].

A top facilitator of confidence is doctors’ recommendation [2,19,32,59], motivating 80% of Chinese [29] and 62% of Americans [13] (compared to 54% if the FDA endorsed the vaccine safety [56]). Opinion of families and friends also played a role [2]. Past inoculation history, including influenzas [1,5,7,9,48] and MMR (Measles, Mumps, and Rubella) vaccines [60], was another proven indicator. Conversely, 40–42% said they are more likely to get a flu shot because of COVID-19 [54,61].

Three international studies investigated healthcare professionals’ attitudes and found similar concerns about vaccine safety and effectiveness and receptivity predictors including previous vaccination history, perceived risk or exposure, and being older, male, or a doctor [7,55,62]. Healthcare workers in Indonesia had greater acceptance than the public (OR: 1.57, 95% CI: 1.12–2.20) [63] while nurses in Hong Kong indicated low intention (40%) [7]. Israeli doctors reported slightly higher self-acceptance (78% vs. 75%), but were less likely to vaccinate their children than the public (60% vs. 70%) [62].

### 3.3. Assessing the Impact of Survey Design

**Figure 2:**
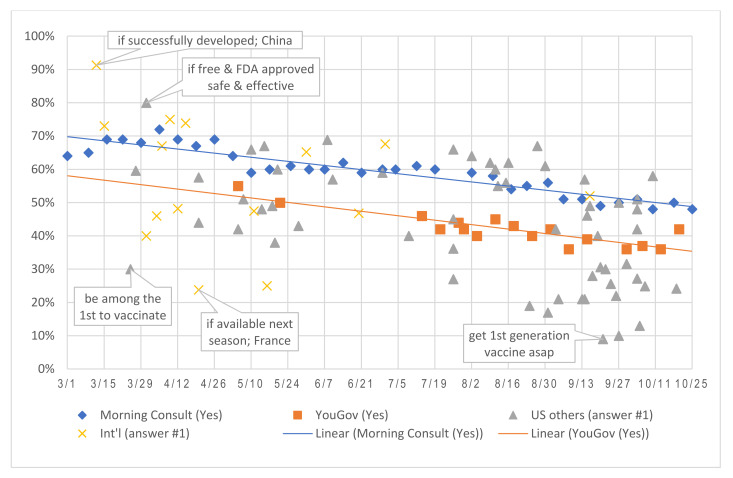
Rates of affirmative responses to a COVID-19 vaccine intention question.

To examine the influence of question framing, Figure 2 plotted the rates of affirmative responses to COVID-19 vaccine intention questions across the past eight months, differentiated US-based and international surveys for comparison. Data from Morning Consult and YouGov series provided strong evidence of the declining receptivity based on consistent questioning. Other surveys, though using varied question wording, showed a similar pattern with few but some exceptions.

Declines in the two longitudinal surveys were almost parallel over the study period, with YouGov’s findings consistently 9–18% lower than Morning Consult’s. YouGov posed the question neutrally as “if and when a coronavirus vaccine becomes available, will you get vaccinated?” Morning Consult worded it slightly differently, “if a vaccine that protects from the coronavirus became available, would you get vaccinated or not?” Similarly, other surveys that reported higher receptivity often framed the question in a more positive way or provided some assurance: e.g., “FDA approved” [40,64], “prevent” [6,65] or “against coronavirus” [18,34,49], “safe and effective” [16,66], “successfully developed” [29], and “recommended for me” [9]. Some surveys tagged additional conditions that triggered higher interest, such as “free” or “at no cost” [6,40,67] and “US-developed” [57]; other conditions heightened hesitancy, including “first generation of vaccine” [45], “as soon as possible” [68,69], and “approved or released before the US election” [70,71,72]. Examples of such effects and outliers were illustrated with annotated speech bubbles in the following graphs.

**Figure 3:**
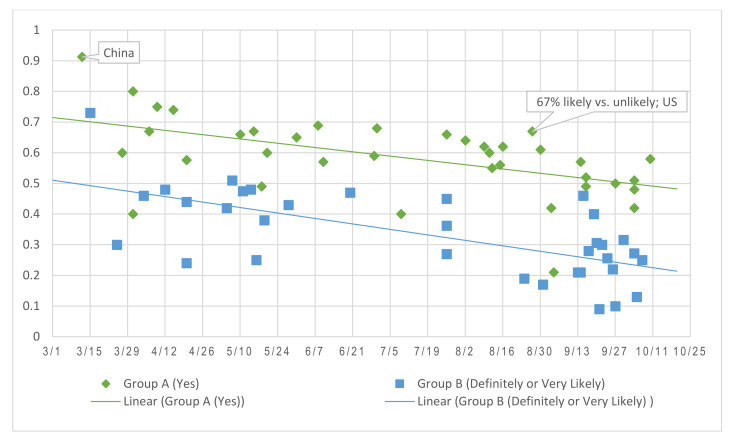
Comparing response differences due to answer option design (percentages of participants chose Yes vs. Definitely/Very Likely to a vaccine intention question) *. * Data points do not include Morning Consults and YouGov to simplify the visual presentation.

Assessing answer choice influence, we separated surveys into Group A with two or three answer options (yes/no/not sure or don’t know) and Group B with four or five options (e.g., very likely/somewhat likely/neutral/somewhat unlikely/very unlikely, definitely/probably/probably not/definitely not). Figure 3 plotted the percentages of participants who picked the first answer in each of the two groups and demonstrated the differences between the two survey designs. Responses were more spread out when there were more options (as in Group B) and thus produced seemingly lower percentages of affirmative answers. Pollsters often combine the results of the first or last two answer categories in writing news articles for more eye-catching headlines, such as “Two in three Americans likely to get coronavirus vaccine [73].” In such case, attention is needed to distinguish and accurately interpret the results.

Further, simply looking at the ratio of people answering yes or likely does not tell the whole story. When answer options included different timings for vaccination, more people chose to wait than get it as soon as possible [54] (e.g., 45% vs. 28% [74]). One survey asked “How likely are you to get a COVID-19 vaccine as soon as it becomes available?” with only two answer choices—likely or unlikely, which by comparison received relatively high (67%) affirmatives [75].

**Figure 4:**
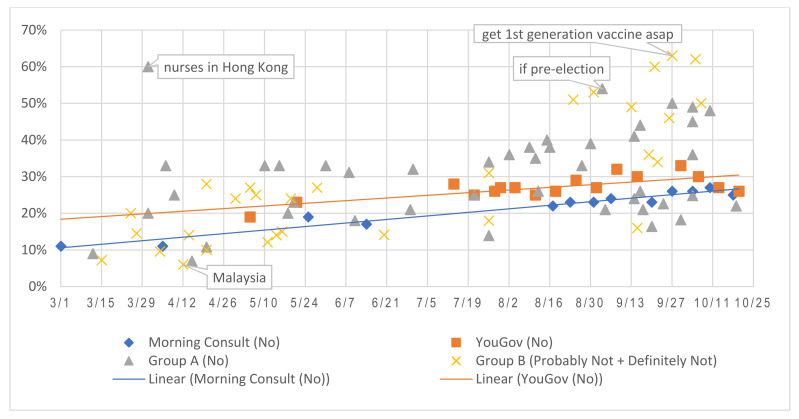
Percentages of expressed refusal or hesitancy to a vaccine intention question.

It is equally important to assess trends of vaccine hesitancy and refusal. Figure 4 illustrated the increasing ratio of respondents indicating low or no intention to vaccinate and the summary table (Appendix A Table A4) documented each answer choice frequency.

### 3.4. Contextual and COVID-19 Factors

Several factors are unique to this pandemic due to the novelties and magnitude of COVID-19 and current highly polarized partisan environment [13,19,76,77,78]. The expedited vaccine development has caused apprehension and distrust [5,10,30,60,79], particularly the Emergency Use Authorization process [53]. While 33% were confident that the FDA will only approve the vaccine if it is safe [51], 41% believed the vaccine will be made available before proven safe and effective [26]. Seventy-five percent worried about the safety of fast-tracking [28]; 11% would be more likely to take a vaccine if Operation Warp Speed suggested it [80].

Hesitancy was manifested in the preference to wait [54]: 60% were unlikely to get the first generation of vaccine [56]; 64% endorsed prioritizing full testing even if delaying availability [57]. Among individual states, 41.6–51% would wait until others have taken it [23,25,58,74,81,82,83]. In China, while 91.3% showed intention to accept, 47.8% would delay until confirmed safe [29]. On the other hand, one international survey found 43% willing to accept less stringent standards [20]; an American survey reported that 59% agreed that providing more people access outweighs the risks of an accelerated process [30].

Three studies analyzed the prevalence and impact of conspiracy theories [84,85,86]; 33% of respondents in the US and 50% in England showed some conspiracy thinking [84,85]. Respondents with higher skepticism had lower perceived risk and trust in government or professionals, and thus higher doubts and objections to vaccination [85,86]. Mainstream news could counter misinformation and utilizing politically conservative outlets and doctor’s communications were suggested for accurate messaging [84,86].

Coinciding with the timing of the US presidential election, many polls included politically oriented questions. Partisan influences were evident, with persistent vaccine attitude gaps between Democrats and Republicans [27,50,76] (80% vs. 48% acceptance [40]; 74% vs. 54% belief in clinical trial importance [42]). The chasm extended to risk perceptions; 42% vs. 19% believed coronavirus is a severe health threat [76]. Conversely, in France, the Far Right parties had higher willingness to vaccinate [10]. Declining trust of information sources and authorities was also observed [24,56,60,87,88]: 50% thought President Trump had influence over FDA decisions [89]; 82% of Democrats and 72% of Republicans worried vaccine approval was driven more by politics than science [75].

In a large multi-national survey, 71.5% would likely get vaccinated and 61.4% would comply if employers suggested doing so [21]. In the US, 65% believed parents should be required to vaccinate [90]; but the rate of refusal grew from 24% to 42.2% in North Carolina and 16.4% to 35.4% in Maine, if it was mandated by the federal government [23,83]. Views varied concerning children: 51% of Americans believed K-12 schools (kindergarten through grade 12) should require COVD-19 vaccines, while 75% of Texans thought the government should require child vaccination for infectious diseases [54,91]. Parents may have different considerations for vaccinating their children than themselves. One survey reported 76.02% vs. 74.38% receptivity and another 45% vs. 36.2% [5,54]; 58% would do so as soon as possible [51], though having chronic illness was a deterrence [19]. Those who indicated refusal for themselves also would not vaccinate their children [48].

## 4. Discussion

This review is the first examining trends of over 100 surveys capturing COVID-19 vaccine receptivity. Although there appear to be consistent declining trends, there have been differences in survey presentations and findings. The deep fissures in American society by income, race, and political affiliation revealed by the pandemic are reflected in vaccine attitudes. Our results showed that vaccine hesitancy is universal across countries, states, and subgroups (including healthcare providers and parents), so are its determinants—perceived disease or outbreak severity, infection risk, and vaccine safety, effectiveness, and necessity. Influenza vaccination history, trust in government, and doctor’s recommendations are important facilitators for vaccine confidence and acceptance. These findings align with previous research on other vaccines [92,93,94,95]. Nonetheless, increasing daily cases and deaths did not prevent double-digit declines in vaccination intention since its highest point in early April [27].

Socioeconomic and racial issues pertaining to health disparity during regular times and other epidemics persist here [96,97]. Minorities, lower income, and less educated individuals are disproportionally more susceptible to COVID-19 [98,99]. Their considerably lower vaccine acceptance requires special attention, including acknowledging the source and addressing the effect of their chronic distrust of health authorities in order to confront the vicious cycle of skepticism and inferior health outcomes. Much of minorities’ reservation or resistance toward medical research and the healthcare system originated from historical events (e.g., unethical experimentation among Blacks in the Tuskegee syphilis study) as well as ongoing perceived bias in clinical interactions and treatments [97,100,101]. Vaccine distribution prioritization should consider these disadvantaged groups as part of the high-risk population, considering their work or underlying health conditions, to improve equity [102].

Men in general are more receptive of COVID-19 vaccines and, as evident in the literature, more inclined to adopt pharmaceutical interventions [103], including vaccination [104,105,106]. Women are more likely to worry about catching coronavirus, concerned about side effects [107], and take protective measures (e.g., masking, handwashing, and social distancing) [108,109]. Such prevention-orientation variance, not limited to gender difference, calls for tailored communications and appeals. Future research could explore whether people perceive higher risk in taking a new vaccine than getting infected with a novel disease.

Often dominating the vaccine discussion in this pandemic are unique expedited development, perceived political interference, and ubiquitous misinformation that have dampened confidence in the rigor of the approval process and the use of the vaccine itself. Trust in authorities has fallen, greater in federal than state or local governments (53% in mid-March to 34% in October) [72]. Major news media believed President Trump’s repeated pre-election promises of a vaccine “within weeks” or by November often “fueled fears” and “heightened concerns” of a rushed process [110,111]. Our polarized electorate mirrors that of the population with large differences across groups. The devastating economic consequences of the pandemic and the heated US presidential election filled with clashing rhetoric have further divided the society along the party line, partitioning people’s opinions in the reality of COVID-19 and the life-saving measure against it. Moreover, the proliferation of conspiracy theories surrounding coronavirus and the vaccines, combined with existing anti-vaccine movement adds another uncertain dimension to vaccine decisions [86,112,113]. Detected misinformation or the spread of “fake news” must be quickly denounced and sources isolated.

Emphasizing transparency and adherence to scientific standards throughout the vaccine development, approval, and distribution processes could restore confidence. In early September, the pharmaceutical industry’s joint pledge to file for emergency authorization only when they have evidence proving the safety and effectiveness in clinical trials [114] boosted pharma’s reputation to 49% positive view in a national poll [115], compared to 32% pre-COVID-19 [116]. The FDA advisory committee’s public meetings with independent experts also provided some reassurance [110]. Second to doctors, Centers for Disease Control and Prevention (CDC) and national public health officials remain the most trusted sources for accurate information (71% and 69%, respectively, though decreased since mid-March) [72]; these figures are significant influencers of people’s health behavior and should be the main communicators in vaccine campaigns to encourage acceptance [117].

The impact of framing and wording choices demonstrated in the comparative analyses offers lessons to guide the urgent development of a critically needed national vaccine campaign and improve future study design supporting continued vaccine hesitancy surveillance [118,119]. For example, posing a question such as, “Would you be willing to get the vaccine to protect yourself and your family?” casts a more positive mindset than asking “how risky do you think it would be to get vaccinated?” Yet, the latter could be turned into an educational opportunity to correct misconception. Though a vaccine could not be distributed without FDA approval, from the surveys many people do not seem to equate a vaccine becoming available to having been approved with proper safety protocols in place. Delineations on such issues could debunk confusion and doubts.

Subgroups with different characteristics and opinions require customized messages, presentations, and channels [120]. It is essential to ensure that intention translates into actual uptake [121]. Furthermore, hesitancy when compared to avoidance or refusal, is a dynamic state that opens the door for persuasion [22,118]. Campaigns targeting those who responded “likely,” “probably,” or “not sure” regarding vaccine intention would be more fruitful than trying to convert those who stated “no” or “definitely not.” People need to believe that a behavior is beneficial, even vital, in order to adopt it [122]. Messages should focus on the safety and efficacy of vaccines as well as clarify the value and necessity of immunization in people’s belief system (e.g., stressing that many vaccines have helped eradiate or control deadly diseases that we are no longer aware of or concerned about because vaccines worked).

Learning from the delayed and conflicting communications about mask wearing and the protest against it, messages tailored to individuals’ disposition (e.g., protecting self or others and family, freedom of choice vs. civic responsibility) would be more effective. Though not explicitly covered in the surveys reviewed, the intricate balance between preserving individual rights and securing population health has generated discords throughout the pandemic, from mask requirement, lockdowns or curfews, to mass vaccination. Several studies underscored the likely resistance mandates may elicit, even among originally receptive groups [23,25,81,83]. Framing vaccination as a smart, purposeful personal decision, emphasizing individual’s autonomy could yield greater results. In addition to traditional media and official websites for disseminating current and accurate information, since social media is a popular source of news as well as misinformation for many [123,124], it should be a key channel in messaging and combating anti-vaccine or conspiracy theories.

Communication strategies could utilize positive cues to action, including encouragement from loved ones and trusted figures such as physicians and religious leaders, sharing personal stories, and peer pressure [125]. Studies also have shown social expectation and portraying anticipated regret from inaction to be potential motivators for vaccination [104,126]. Furthermore, accompanying the rollouts of vaccines with short supply and complex delivery requirements (e.g., low-temperature storage and double dosage), campaign objectives should instill confidence not just in the safety of the medical intervention but also in the manufacturing, transportation, access, and equitable distribution to alleviate concerns or distrust.

This review is subject to limitations. Studies retrieved from the scholarly databases may not provide the most up-to-date public opinions due to the review and publication processes. Though Google search is less customary for systematic reviews, research guides suggested it as a gray literature source and suitable in locating surveys for this review [127,128,129]. The inclusion of studies was not exhaustive (with mostly US-based surveys), but covered a large number of major polls and important factors for a comprehensive picture of the trends. Future research would benefit from qualitative inquiries to allow for elaborations on non-pre-defined factors. Longitudinal studies could re-poll the same participants to detect triggers for attitude changes.

Caution should be taken in interpreting and using the results since intention or survey responses may not directly predict future behavior [130]. Moreover, opinions may change, especially amid the raging pandemic. Continued vaccine receptivity tracking could reveal whether the reported clinical trials incidents or outcomes and subsequent introductions of vaccines or new treatments would further change people’s minds about getting vaccinated.

## 5. Conclusions

Vaccine hesitancy is an imminent threat in the battle against COVID-19 because achieving herd immunity depends on the efficacy of the vaccine itself and the population’s willingness to accept it. This review offered a sweeping examination of the evolving vaccine attitudes since the early stage of the pandemic to inform policy makers and public health professionals in campaign planning and communications. Consistent with the literature, demographic and socioeconomic divides in receptivity are present in these surveys and the partisan nature of some indicators is unprecedented. Multiple factors, including perceived disease risk and vaccine safety concern as well as question presentation, could influence responses and ultimately actions. The power of words and framing illustrated in this review helps shed light on strategic communication for motivating positive, collective pandemic response.

On-going campaign content adjustments and monitoring responses should not be overlooked. Once vaccination starts, the likely decrease in new COVID-19 cases needs to be accurately highlighted as the outcome of vaccine uptake rather than being interpreted as lessened risk, something that could reduce the perceived need for vaccination.

## Appendix A. Literature Search Strategy

**Table A1:** Database: PubMed (Search Date: 20 October 2020).

Set #		Results
1	“coronavirus”[MeSH Terms] OR “coronavirus”[All Fields] OR “coronaviruses”[All Fields] OR “covid 19”[All Fields] OR “SARS-2”[All Fields] OR “severe acute respiratory syndrome coronavirus 2”[All Fields] OR “severe acute respiratory syndrome coronavirus 2”[Supplementary Concept] OR “ncov”[All Fields] OR “2019 ncov”[All Fields] OR “sars cov 2”[All Fields] OR ((“coronavirus”[All Fields] OR “cov”[All Fields]) AND 2019/11/01:3000[Date—Publication])	80,867
2	“vaccines”[MeSH Terms] OR “vaccin”[All Fields] OR “vaccination”[MeSH Terms] OR “vaccination”[All Fields] OR “vaccinable”[All Fields] OR “vaccinal”[All Fields] OR “vaccinate”[All Fields] OR “vaccinated”[All Fields] OR “vaccinates”[All Fields] OR “vaccinating”[All Fields] OR “vaccinations”[All Fields] OR “vaccination’s”[All Fields] OR “vaccinator”[All Fields] OR “vaccinators”[All Fields] OR “vaccine s”[All Fields] OR “vaccined”[All Fields] OR “vaccines”[All Fields] OR “vaccine”[All Fields] OR “vaccins”[All Fields] OR “vaccin”[Supplementary Concept]	394,922
3	“surveys and questionnaires”[MeSH Terms] OR “survey”[All Fields] OR “surveys”[All Fields] OR “survey’s”[All Fields] OR “surveyed”[All Fields] OR “surveying”[All Fields] OR (“surveys”[All Fields] AND “questionnaires”[All Fields]) OR “surveys and questionnaires”[All Fields] OR (“questionnair”[All Fields] OR “questionnaire’s”[All Fields] OR “surveys and questionnaires”[MeSH Terms] OR (“surveys”[All Fields] AND “questionnaires”[All Fields]) OR “surveys and questionnaires”[All Fields] OR “questionnaire”[All Fields] OR “questionnaires”[All Fields]) OR “poll”[All Fields]	1,705,583
4	1 AND 2 AND 3	298
5	Filters: from 2020/1/1	216

**Table A2:** Database: Embase (Search Date: 20 October 2020).

Set #		Results
1	‘covid 19’ OR ‘covid 19’/exp OR coronavirus OR coronavirus/exp OR ‘2019 ncov’ OR ‘2019 ncov’/exp OR ‘severe acute respiratory syndrome coronavirus 2’ OR ‘severe acute respiratory syndrome coronavirus 2’/exp OR ‘SARS-COV 2’ OR ‘SARS-COV 2′/exp	86,215
2	vaccine OR vaccine/exp OR vaccination OR vaccination/exp OR immunization OR immunization/exp	603,926
3	survey OR survey/exp OR questionnaire OR questionnaire/exp OR poll OR poll/exp	2,177,885
4	1 AND 2 AND 3	306
	AND [2020–2020]/py	173

**Table A3:** Database: PsycINFO * (Search Date: 20 October 2020).

Set #		Results
1	covid-19 OR coronavirus OR 2019-ncov OR sars-cov-2 OR cov-19	2162
2	vaccine OR vaccines OR vaccination OR immunization OR immunizations	9407
3	survey OR questionnaire OR poll	709,844
4	1 AND 2 AND 3	6

* Applied related words and equivalent subjects, searched within the full text of the articles.

**Table A4:** Summary Table of Survey Design and Findings of Selected Studies on COVID-19 Vaccine Receptiveness *.

					Response Percentages					
Survey Dates (# in Series)	Author or Organization [int’l Study]	Sample Size (*n*) ^†^	Main Question	Answer Choices	Yes, Very Likely	Some-what Likely, Probably	Not Sure, Don’t Know	Some-what Unlikely, Probably Not	No, Not at All Likely, Very Unlikely	Key Findings and Relevant Factors
2/28–3/1 (1 of 33)	Morning Consult [131]	2020	If a vaccine that protects from the coronavirus became available, would you get vaccinated or not?	Yes/don’t know/no	64%		25%		11%	More likely to accept COVID vaccine: male, 18–29, Liberal, post-grad, higher income, work in government
March	Wang et al. [29] [China]	2058	If a COVID-19 vaccine is successfully developed and approved for listing in the future, would you accept vaccination?	Yes/no	91%				9%	80% consider doctors’ recommendation, 60% said price important; 52% would get as soon as possible (ASAP) and 48% wait ‘til confirmed safe; 64% no preference for domestic vs. imported vaccine. More likely: male, married, high risk, pandemic large impact, convenient
March	Abdelhafiz et al. [17] [Egypt]	559 ^†^	If there is an available vaccine for the virus, I am willing to get it.	Strongly agree/ agree/neither agree or isagree/disagree/ strongly disagree	73%	15.6%	4%	2%	5%	Younger group have higher COVID knowledge, male-female similar; 86% view COVID dangerous, 16.8% think media coverage exaggerated; 26.8% believe COVID designed as biological weapon; overall positive toward preventative measures
3/24–3/25	Morning Consult, NBC LX [132]	2200	If a vaccine for coronavirus—aka COVID-19—became available, how quickly would you get vaccinated, if you were to get vaccinated at all?	Among the first/ in the middle/ I don’t know/ among the last/ I would not get vaccinated	30%	34%	15%	11	9%	Asked if vaccine should be required, free, development accelerated skipping clinical trials, benefits outweigh risks. 74% likely to get if passes trials; 30% “be in a rush” to get FDA-approved vaccine. 66% believe vaccine more effective than social distancing to control spread
3/17–3/27	Romer et al. [86]	1050	If there were a vaccine that protected you from getting the coronavirus, how likely, if at all, would you be to decide to be vaccinated?	Very likely/likely/ not likely/not at all likely	60%			14.5% (3 + 4)		Assessed conspiracy theory impact: hesitancy increase predicted by earlier beliefs (“pharma created coronavirus to increase sales”, “MMR (measles, mumps, and rubella) vaccine can cause neurological disorders”). Conservative and social media use positively related to conspiracy thinking
2/26–3/31	Wang et al. [7] [Hong Kong]	806 ^†^	Asked whether or not they intended to accept COVID vaccination when it is available.	Intend to accept/ not intend to accept (undecided)	40%				60%	Nurses. More likely: in public sector, 30–39, w/chronic condition, infection likelihood. Refusal reasons: efficacy/safety concerns, believed unnecessary, no time. Past flu vaccination strong predictor and lessened high hesitancy. Increased flu shot intent b/c COVID.
3/24–3/31	Thunstrom et al. [48]	3133	Would anyone in your family get the coronavirus vaccine (conditions: FDA approved, 60% effective, available today, free)?	Would/would not	80%				20%	Compared scenarios. Concerns: vaccine newness, side effects/efficacy. Less likely: female, believe in God, not had flu shots. Inconsistent risk messages (White House vs. Centers for Disease Control and Prevention (CDC)) deterrence. Those refused also won’t vaccinate their child.
3/28–4/4	Ali et al. [16] [Bahrain]	5677 ^†^	Suppose that a safe and effective coronavirus vaccine was available today. How likely are you to get yourself vaccinated?	Very likely/ somewhat likely/ neutral/ somewhat unlikely/ very unlikely	46%	26.20%	18%	6.60%	3%	Posted on social media. Most knowledgeable of COVID symptoms and preventive measures. More likely: younger (only 7.5% 18–34 year unlikely vs. 14.6% 35+), male, work/study in healthcare (51.7% very likely vs. 44.2% public)—also higher perceived infection risk. Main COVID info sources: social media and World Health Organization (WHO).
3/25–4/6	Harapan et al. [63] [Indonesia]	1068	Whether they would be vaccinated with a new COVID-19 vaccine for each scenario (50% or 95% effective).	Yes/no	67% (@50% efficacy)				33%	Question premise: tested clinically, free and optional, 5% chance side effect. Compared scenarios: 93% accept @95% efficacy. More likely @95%: healthcare workers (HCW) (aOR: 2.01) and higher perceived risk (aOR: 2.21); retired less likely. If @50%: HCW (aOR: 1.57).
3/26–4/9	Dror et al. [62] [Israel]	1941	Would you vaccine yourself for COVID-19?	Yes/no	75%				25%	Compared w/HCW. 70% public and Drs had safety concern. More likely: male, higher perceived risk, doctors (78%), lost job due to COVID (96%). 70% public likely to vaccinate their child, vs. 60% Drs and 55% nurses.
4/3–4/12	Wong et al. [34] [Malaysia]	1159	If a vaccine against COVID-19 infection is available in the market, would you take it?	Definitely/ probably/possibly/probably not/ definitely not	48%	29.80%	16%	3.30%	2.40%	Health Belief Model: benefit belief (OR: 2.51) and feel less worried having vaccine (OR: 2.19). High perceived infection risk and barriers (cost, if halal, inadequate info, safety/efficacy concerns). Avg willingness to pay US$30.66; 74.3% will wait to get vaccinated.
4/13–4/14	Earnshaw et al. [84]	845	When a vaccine becomes available for the coronavirus, how likely are you to get it?	Very likely/ somewhat likely/ likely/ unlikely/ not at all likely	85.8% (1 + 2 + 3)			14% (4 + 5)		Less likely: women, less edu, believed conspiracies (3.9 X less). 33% w/conspiracy beliefs: younger, Black and minorities, college edu, less COVID knowledge and policy support, medical mistrust, use social media. Drs are most trusted info source (90%).
4/2–4/15	Neumann-Bohme et al. [22] [7 European countries]	7664	If available, would you be willing to get vaccinated? (not exact wording)	Yes/unsure/no	74%		19%		7%	7 nations, willingness varied: France 62%—Denmark 80%; opposition 10% in Germany and France; largest unsure: France 28%. More likely: 55+, male; women reject 2X men. 55% concerned about side effects.
2/26–4/20	Detoc et al. [18] [France]	2512 ^†^	If a vaccine against the new coronavirus was available for next season, would you get vaccinated?	Yes, certainly/ yes, possibly/I don’t know/No, possibly/ Definitely no	24%	53.8%	12.1%	6.4%	3.9%	More likely: men, older, fear COVID, perceived risk, HCW (81.5% vs. 73.7%). 74.7% fear COVID, 65.2% self-considered at risk. 47.6% would participate in clinical trials: older, men, HCW, higher perceived risk
4/16–4/20	Fisher et al. [1]	1000	When a vaccine for the coronavirus becomes available, will you get vaccinated?	Yes/not sure/no	57.6%		31.6%		10.8%	Less likely: younger, female, Black/Hispanic, lower income/edu, larger household, rural, not had flu shot. Qualitative inquiry of reasons. Hesitant: specific vaccine concerns, antivaccine attitudes, not trusting entitles involved in vaccine dissemination.
4/18–4/20(1st of 2)	The Harris Poll [133]	2029	How likely are you to get a COVID-19 vaccine as soon as it becomes available?	Very likely/ somewhat likely/ not very likely/ not at all likely	44%	29%		16%	12%	(Part of longitudinal study since 3/14 on other topics) 57% would likely get flu shot; 73% for COVID: 86% in Michigan, 72% in NY. Parent vs. non-parent similar likelihood (75% and 72%)
4/7–5/4	Ward et al. [10] [France]	5018	whether they would agree to get vaccinated if a vaccine against the COVID-19 was available	Certainly/ probably/ probably not/ certainly not	76% (1 + 2)			16.1%	8%	Asked partisan preferences, COVID concerns and diagnosis. More likely: Far Right parties, females, <35 year, high school edu. Refusal reasons: against vaccination in general, too rushed, thought useless.
4/29–5/5 (1 of 2)	Pew Research Center [30]	10,957	If a vaccine were available today, I definitely/ probably ____ get it	Definitely/ probably/ probably not/ definitely not (no response)	42%	30%		16%	11%	More likely: male, Boomer+, postgrad, Democrat, Catholic. 74% White and Hispanic, 91% Asian, 54% Black; 74% Dem vs. 54% Rep said clinical trials important. 59% said benefits of allowing more people access outweigh risks.
5/4–5/5 (1 of 17)	YouGov, Yahoo News [40]	1573	If and when a coronavirus vaccine becomes available, will you get vaccinated?	Yes/not sure/no	55%		26%		19%	When would be available: 51% believed in 2021, 24% in 2020. More likely: 18–29, Hispanic (62%), Democrat, suburb, higher income. Male 56% vs. female 54%.
5/6–5/7 (1 of 2)	ABC News, Ipsos [66]	532	If a safe and effective coronavirus vaccine is developed, how likely would you be to get vaccinated?	Very/somewhat/ not very/not at all (no answer)	51%	24%		14%	11%	77% concerned self or someone they know will be infected (66% in March). 64% view opening now not worth it b/c would lead to more deaths
5/10–5/16 (1 of 3)	CNN/SSRS [134]	1112	If a vaccine to prevent coronavirus infection was widely available at a low cost, would you, personally, try to get that vaccine, or not?	Yes/no/ no opinion	66%				33%	More likely: 65+, Trump disapproving, college grad, Democrat. 36% feel more comfortable if vaccine existed; 41% more comfortable returning to regular routine.
5/4–5/11	Freeman et al. [85] and compliance with government guidelines in England [England]	2501	Take a COVID-19 vaccine if offered?	Definitely/ probably/possibly/probably not/ definitely not	47.5%	22.1%	18.4%	7.3%	4.8%	50% endorse conspiracy beliefs. Higher conspiracy thinking: less adherence to all gov guidelines, less willingness to take tests or vaccine, more likely to share opinions, also connect to other mistrust.
May	Reiter et al. [2]	2006 ^†^	How willing would you be to get the COVID-19 vaccine if it was free or covered by health insurance?	Definitely not willing/probably not willing/ not sure/ probably willing/ definitely willing	48%	21%	17%	5%	9%	Less likely: Black, low income, uninsured, conservative. 35% would pay $50+. Provider recommendation, perceived risk and severity, vaccine effectiveness (or harms) correlated w/acceptability
May	Malik et al. [9]	672	If a vaccine becomes available and is recommended for me, I would get it.	Agree/strongly agree/neutral/disagree/strongly disagree	67%				33%	Compared US regions/states, between flu and COVID vaccine acceptance. Region 2-NY lowest (thought epicenter). Highest rates: ND, SD, MN, MT, WY, UT, CO. Less likely for both vaccines: Blacks, lower income/edu
May	Graffigna et al. [8] [Italy]	1004	Willingness to vaccinate against COVID-19 whenever the vaccine is available.	Very likely/ somewhat likely/ likely/unlikely/not at all likely	25%	33%	26%	7%	8%	Path Model: health engagement positively related to intention, mediated by general vaccine attitude, perceived severity and susceptibility; invariant across gender, parallel w/other nations (France, US, Poland)
5/14–5/18	The Associated Press, Univ of Chicago, AP-NORC Center for Public Affairs Research [49]	1056	If a vaccine against the coronavirus becomes available, do you plan to get vaccinated?	Yes/not sure/no	49%		31%		20%	Top concern: side effect. 55% of those worried self/family get infected would vaccinate. 61% believe it will be available in 2021. Acceptance reasons: protect self and family, feel safe around others, best way to avoid getting seriously ill.
5/13–5/19	Reuters, IPSOS [135]	4428	How interested would you be in getting a coronavirus/COVID-19 vaccine, if at all?	Very likely/ somewhat likely/ unsure/ not very likely/ not at all likely	38%	27%	11%	10%	14%	48% worried vaccine coming out quickly, 42% concerned risks; most be more interested if there was large scientific study to confirm safety. Refusal reasons: newness, risks outweighing benefits. More interest if developed in US vs. Europe or China
5/17–5/20 (1 of 2)	Beacon Research, Shaw and Co Research, Fox News [136]	1207	Do you plan to get a vaccine shot against coronavirus when a vaccine becomes available, or not?	Yes/don’t know/no	60%		16%		23%	61% “very” concerned about spread of coronavirus in US (also compared swine flu vaccine opinions in 2009).
5/23–5/28	Washington Post, ABC News [67]	1001	If a vaccine that protected you from the coronavirus was available for free to everyone who wanted it, would you get it?	Definitely/ probably/ probably not/ definitely not	43%	28%		12%	15%	Refusal reasons: lack of trust, thought unnecessary. 57% believed more important to control pandemic even if it hurts the economy
3/26–5/31	Goldman et al. [19] [Canada, Israel, Japan, Spain, Switzerland, US]	1541 ^†^	There is no vaccine/ immunization currently available for Coronavirus (COVID-19). If a vaccine was available today, would you give it to your child?	Yes/no	65%				33%	Asked caregiver (mostly parents) willingness to vaccinate their child; w/follow-up open questions. More likely: older age of children and caregiver, up-to-date on vaccination, no chronic illness, father surveyed, more concerned about child than self having COVID. Intent: protect child; common refusal b/c vaccine novelty
5/28–6/8	Callaghan et al. [42]	5009	Scientists around the world are working on developing a vaccine to protect individuals against the coronavirus. If a vaccine is developed, would you pursue getting vaccinated for the coronavirus?	Yes/no	68.9%				31.1%	Compared and hypothesized hesitancy reasons across subgroups. Blacks 40% more likely to refuse b/c lack of trust in safety, efficacy, and financial resources. Less likely: women, conservatives, those see vaccines unimportant/ineffective, Trump voters, religious. Those been tested for COVID 68% less likely to refuse vaccination.
5/29–6/10	Tufts Univ Research Group on Equity in Health, Wealth and Civic Engagement [33]	1267	If a vaccine were available today, would you be willing to get it? (not exact wording)	Yes/don’t know/no	57%		24%		18%	Examined hesitation in equity context. More likely: Whites, Hispanics, Democrats, more formal edu, higher income. Further widened “the gap in health outcomes”
6/16–6/20	Lazarus et al. [21] [19 countries]	13,426	If a COVID-19 vaccine is proven safe and effective and is available to me, I will take it.	Completely agree/ somewhat agree/ neutral/ somewhat disagree/ completely disagree	47%	24.7%		6.1%	8%	Compare globally. Highest: China 88.6%, Brazil 85.4%, S Africa 81.6% (US 75%); lowest—Russia 54.9%. Highest refusal: Russia 27.3%. 61.4% would follow employer recommendations. More likely: trust gov, higher income/edu, older (18–24 least), women slightly more, higher national case and mortality rates. Younger more likely to accept if employer suggest.
6/19–6/29(1 of 2)	YouGov, Univ of Texas [91]	1200	If a vaccine to prevent coronavirus infection were widely available at a low cost, would you try to get that vaccine, or not?	Yes/no opinion/no	59%		20%		21%	Texas. 75% believe gov should require parents to have their children vaccinated against infectious diseases, 14% disagreed
3/26–6/30	Goldman et al. [20] [Canada, Israel, Japan, Spain, Switzerland, US]	2557 ^†^	Would you vaccinate your child against COVID-19 if a vaccine existed today?	Yes/no	68%				32%	43% willing to accept less strict standards of development and approval; More likely to vaccinate their child: if father surveyed (compared to mother), child have followed recommended vaccination schedule, concerned about having COVID-19
7/9	Kreps et al. [53]	1971	How likely to receive a (scenario 1) vaccine with 50% efficacy, a 1-year protection duration, was approved under an FDA EUA, and developed in China?	(model estimated willingness)	40%					Compare scenarios. More likely: increased efficacy and protection duration, decreased in adverse effect; had regular flu shots, favorable attitudes toward pharma. Less willing: female, Black, personal contact w/someone tested positive, believe pandemic would worsen; if FDA EUA or non-US-made; if endorsed by Trump (vs. CDC/WHO).
7/10–7/26 (1 of 2)	COVID-19 Consortium for Understanding the Public’s Policy Preferences Across States, PureSpectrum [24]	19,058	If a vaccine against COVID-19 was available to you, how likely would you be to get vaccinated?	Extremely likely/somewhat likely/neither likely nor unlikely/somewhat likely/extremely unlikely	45%	21%	15%	6%	12%	Compare 50 states @30–50–70–90% effectiveness. Top factors: safety, effectiveness, side effects, protect self/family, Dr. recommend. 66% would vaccinate their children. Associated w/mask-wearing. >60%: AL, AR, LA, MS, MO, OH, OK, SD, WV, WY. 70%+: AZ, CA, Iowa, MD, MA, MN, ND, NY, RI, UT, WA, DC. More likely: Asian, male, 65+, Democrat
7/20–7/26 (1 of 6)	Gallup [40]	7632	If an FDA-approved vaccine to prevent coronavirus/ COVID-19 was available right now at no cost, would you agree to be vaccinated?	Yes/no	66%				34%	Compared ethnicities “whites” and “non-whites”: 67% vs. 59%. 83% Democrats vs. 46% Republicans; men (67%) and women (65%) relatively equally likely. Least likely: middle age (vs. 76% 18–29 and 70% senior)
7/24–7/26	Lending Tree, Value Penguin [54]	1010	Once the coronavirus vaccine is available to the public, do you plan to get vaccinated?	Yes/only if covered by insurance/ depends on circumstances/definitely not (I don’t know)	36.2%	17.1% (if with insurance)	25.9% (depends)		13.9%	42% would wait at least weeks before getting vaccinated. 51% believe public schools should require; 45% parents would definitely vaccinate their child. ~40% more likely to get flu shot b/c of COVID
7/24–7/26	Politico, Morning Consult [57]	1997	If the US were to develop a vaccine for the coronavirus that was available to Americans, how quickly would you get vaccinated, if you were to get vaccinated at all?	Among the first/in the middle/ I don’t know/ among the last/ I would not get vaccinated	27%	31%	11%	14%	17%	23% would decline if China-made vs. 17% if US-made, especially among Trump supporters. 64% believe US should prioritize fully testing even if delaying availability and continued COVID spread. 44% trust Biden more to oversee development (vs. 33% Trump)
7/24–7/27	Axios, Ipsos-Knowledge Panel [137]	1076	How much of a risk to your health and well-being do you think the following activities are right now—taking the 1st generation COVID-19 vaccine as soon as it’s available?	No risk/ small risk/ moderate risk/ large risk	8%	29%		43%	19%	63% wear mask at all time, 24% sometimes. 69% thought participating in vaccine trial moderate or large risk, 71% thought sending child to school in fall risky. (Many questions on how things/life have changed, social distancing, work/business closing, access to food and healthcare, trust in public figures and institutions/gov.)
8/3–8/11 (1 of 2)	NPR and PBS NewsHour, The Marist Poll [138]	1261	If a vaccine for coronavirus is made available to you, will you choose to be vaccinated or not?	Yes/not sure/no	60%		5%		35%	More likely: Democrat, college degree, 18–29 and 60+; similar percentages compared to 2009 H1N1 vaccine willingness
8/9–8/12 (2 of 2)	Beacon Research, Shaw and Co Research, Fox News [139]	1000	Do you plan to get a vaccine shot against coronavirus when a vaccine becomes available, or not?	Yes/don’t know/no	55%		20%		26%	Declined willingness from May to August; higher rates compared to earlier swine flu vaccination opinions
8/12–8/15 (2 of 3)	CNN/SSRS [140]	1108	If a vaccine to prevent coronavirus infection were widely available at a low cost, would you, personally, try to get that vaccine, or not?	Yes/no	56%				40%	40% thought worst of COVID is behind us, 55% though yet to come. 68% felt the way US respond to COVID embarrassed (other choice “proud” 28%). Willingness decreased since May; 62% confident that ongoing trials properly balancing safety and speed
8/21–8/24 (1 of 5)	Ipsos/Axois [45]	1084	How likely, if at all, are you to get the first generation COVID-19 vaccine, as soon as it’s available?	Very likely/ somewhat likely/ not very likely/ not at al likely (no answer)	19%	29%		22%	29%	76% social distanced (staying home avoided others) the past week; 68% and 22% wear mask all the time or sometimes; 54% and 37% keep 6-ft from people all the time or sometimes
8/7–8/26 (2 of 2)	COVID-19 Consortium for Understanding Public’s Policy Preferences Across States, Pure Spectrum [37]	21,196	If a vaccine against COVID-19 was available to you, how likely would you be to get vaccinated?	Extremely likely/ somewhat likely/ neither likely nor unlikely/ somewhat likely/ extremely unlikely	59% (1 + 2)					Trust were lower than in April for every institution/figure (Biden, Trump, CDC, Fauci, News media, social media, state gov, police, etc); trust scientists/ researchers much more than president. 73% Democrats who trust Trump and 84% of Republicans who trust Biden would vaccinate their children.
8/25–8/27 (1 of 2)	STAT/The Harris Poll [75]	2067	How likely are you to get a COVID-19 vaccine as soon as it becomes available?	Likely/unlikely	67%				33%	82% Democrats worry vaccine approval more driven by politics than science (vs. 72% Republicans). 46% trust president or WH for accurate COVID info; 68% confident FDA will only endorse a vaccine that is safe
8/28–9/3	Kaiser Family Foundation [141]	1199	If a coronavirus vaccine was approved by the U.S. FDA before the presidential election in November and was available for free to everyone who wanted it, do you think you would want to get vaccinated, or not?	Yes/don’t know/no	42%		4%		54%	62% very/somewhat worried FDA will rush to approve without making sure it’s safe and effective due to political pressure from Trump administration; 81% do not think vaccine will be widely available before election
9/2–9/4	YouGov, CBS News [68]	2493	If a coronavirus vaccine became available this year, at no cost to you, would you…?	Get one as soon as possible/consider one/never get one	21%		58%		21%	75% think president (whoever) should publicly take vaccine to show it is safe; White Democrats 2x more likely than Black. 65% believe vaccine announced this year would be rushed or not had enough testing
9/8–9/13 (2 of 2)	Pew Research Center [47]	10,093	Asked if they would get a COVID 19 vaccine if it were available today.	Definitely/ probably/probably not/ definitely not	21%	30%		25%	24%	72% Asian, 56% Hispanic, 52% White, 32% Black; 44% Rep vs. 58% Dem (72% in May; “definitely” dropped 42% to 21%). 76% concern about side effects—major reason; 77% thought likely it will be approved before fully known safe and effective; 78% concern moving too fast vs. 20% too slow.
9/11–9/14	Univ of Chicago School of Public Policy, AP-NORC Center for Public Affairs Research [142]	1053	If a vaccine against the coronavirus becomes available, do you plan to get vaccinated?	Yes/no	57%				41%	58% said US should keep any vaccine it develops for US first vs. 39% believe should make available to others. 52% would get if US-made; 46% would take non-US-developed. 75% Democrat (vs. 39% Republican) thought WHO should have major role in vaccine development
9/11–9/14 (1 of 6)	Suffolk Univ, USA Today [23]	500	When a federally approved COVID-19 vaccine is available, will you…	Take it as soon as you can/ wait awhile until others have taken it/undecided/ not take it	21%	51% (wait)	4%		24%	North Carolina. 48.8% would vaccinate If mandated by federal gov (42.2% would not, 8.6% undecided)
Sep-tember	Brigham Young Univ. [5]	316	How do you feel about the following statement: I am likely to be vaccinated when a vaccine for COVID-19 becomes available.	Strongly agree/ agree/ neither agree or disagree/disagree/strongly disagree	46%	22%	16%	7.00%	9%	Compared scenarios: available timing, @50–75–99% effectiveness, frequency needed. 66% would get if available in 30 days, 74.38% if 6 months; 6–12 months testing be more comfortable. 45.5% concern over safety; more felt comfortable if US-made than other locations. Income/edu and insurance satisfaction positively correlated with intent
9/11–9/16	Grech et al. [55] [Malta]	1002	Based on this info (describe 3-phase development for efficacy and safety). The COVID vaccine that will arrive in Malta will have gone through these Phases and will be approved and licensed, how likely are you to take the COVID-19 vaccine?	Likely/ undecided/unlikely	52%		22%		26%	Surveyed HCWs. More likely: male (64% vs. 45% female), oldest group, doctors. Hesitancy for influenza vaccine: safety, perceived low disease risk, low priority, access, general anti-vaccine
9/11–9/16 (2 of 2)	NPR and PBS NewsHour, The Marist Poll [143]	1152	If a vaccine for the coronavirus is made available to you, will you choose to be vaccinated or not?	Yes/unsure/no	49%		7%		44%	Declined confidence (60% in August); 13% drop in Independents and 10% Republicans. More likely: Democrat, higher income, college grad, White, over 74, suburban area. 52% were likely to get H1N1 vaccine in 2009.
9/14–9/17	Selzer and Co, DesMoines Register [74]	803	When a federally approved vaccine is available, will you take it as soon as you can, wait awhile until others have taken it, or not take the vaccine?	As soon as possible/ wait until others have taken it/ not sure/ not take it	28%	45% (wait)	6%		21%	Iowa. 45% plan to wait until others have taken it. 6% Democrats won’t take vs. 28% Republicans. Quote from respondent: “I’m 76 years old. I sure don’t want to get this virus. I’m figuring it’s going to be OK. It may not be the best vaccine, but I think it’ll be good.”
9/18–9/19 (2 of 2)	ABC News, Ipsos [73,144]	528	If a safe and effective coronavirus vaccine is developed, how likely would you be to get vaccinated?	Very likely/ somewhat likely/ likely/unlikely/not at all likely	40%	24%		19%	17%	Majority have confidence in Fauci, CDC, WHO, FDA, HHS to confirm safe and effective; 41% confident in Biden and 27% in Trump, 62% in Fauci.
9/18–9/22	Ipsos/Newsy [64]	2010	Once a COVID-19 vaccine has been developed, if it were approved for use by the FDA, how interested would you be in getting the vaccine?	Very likely/ somewhat likely/ likely/unlikely/not at all likely	30%	26%		14%	20%	55% say pandemic made them more likely to support increased federal funding for vaccine development and testing, 48% more likely to support Medicare for all (21% less likely)
9/24–9/26 (2 of 2)	The Harris Poll [51]	1971	How likely are you to get a COVID-19 vaccine as soon as it becomes available?	Very likely/ somewhat likely/ likely/unlikely/ not at all likely	22%	31%		25%	21%	58% would vaccinate kids ASAP. 61% say should only made available abroad once US orders delivered. 42% confident gov approval not motivated by politics; 79% concerned over safety. 33% confident FDA will only approve if safe; 46% say US is prepared to deliver; 47% would use foreign-made. 45% will get flu shot
9/14–9/27 (6 of 6)	Gallup [12,40]	2730	If an FDA-approved vaccine to prevent coronavirus/ COVID-19 was available right now at no cost, would you agree to be vaccinated?	Yes/no	50%				50%	53% Democrats vs. 47% Republicans; 56% men and 44% women; 62% ages 18–34, 44% ages 35–54. Overall observed decline from last survey in August (series started in July)
9/25–10/4 (2 of 2)	YouGov, Univ of Texas [26]	1200	If a vaccine to prevent coronavirus infection were widely available at a low cost, would you try to get that vaccine, or not?	Yes/no opinion/no	42%		21%		36%	Texas. 41% believe COVID vaccine will be made available before proven safe; decline in vaccine willingness since June survey
9/30–10/4	Goucher College [43,145]	1002	If an FDA-approved vaccine to prevent coronavirus was available right now at no cost, would you agree to be vaccinated?	Yes/don’t know/no	48%		2%		49%	Maryland. 69% very or somewhat concerned about self/family contracting COVID. 40% though worst is yet to com. 23% thought reopened too quickly and 58% thought about right. Black more likely to distrust a potential vaccine.
10/1–10/4 (3 of 3)	CNN/SSRS [65]	1205	If a vaccine to prevent coronavirus infection were widely available at a low cost, would you, personally, try to get that vaccine, or not?	Yes/no	51%				45%	Willingness declined since July; shift in willingness for Democrats but Republicans/Trump supporters have remained consistent at 41%. 61% say somewhat-very confident that ongoing trials properly balancing speed and safety.
10/1–10/5 (5 of 5)	Ipsos/Axois [72]	1004	How likely, if at all, are you to get the first generation COVID-19 vaccine, as soon as it’s available?	Very likely/ somewhat likely/ likely/unlikely/ not at all likely	13%	25%		31%	31%	30% likely to get 1st gen ASAP, 55% if it has been on the market for months, 65% if been proven safe/effective by public health officials; 18% likely to get if released before election. 26% would get 1st G if they were paid $100 incentive,33% if paid $500, 45% if paid $1000 (54% not likely).
9/24–10/7	YouGov, St. Louis Univ [146]	931	If the following FDA approved vaccines were available today for free, you would get it?	Definitely/ probably/ probably not/ definitely not	25%	26%		24%	26%	Missouri. Higher trust in CDC, Missouri Department of Health and local public health departments vs. FDA; more trust in FDA in Democrats (82% vs. 66%). Democratic 15% more likely to get the vaccine.
10/7–10/10 (2 of 2)	STAT/The Harris Poll [147]	2050	How likely are you to get a COVID-19 vaccine as soon as it becomes available?	Likely/unlikely	58%				48%	Declined from 69% in August. 59% Whites and 43% Blacks. 40% more likely to get vaccine once Trump tested positive for COVID, 41% said their opinions had not changed
10/16–10/18 (33 of 33)	Morning Consult [31]	2200	If a vaccine that protects from the coronavirus became available, would you get vaccinated or not?	Yes/don’t know/no	50%		25%		25%	55% (64% Blacks) very and 28% somewhat concerned about coronavirus; 56% very and 26% somewhat believed mask effective in preventing spread (older age stronger belief). 26% had family/friend tested positive; 15% know someone personally died from COVID
10/15–10/19 (6 of 6)	Suffolk Univ, USA Today [82]	500	When a federally approved COVID-19 vaccine is available, will you…	Take it as soon as you can/wait awhile until others have taken it/undecided/ not take it (refuse to answer)	24.2%	45.5% (wait)	7.8%		22.2%	Pennsylvania. 45.6% get most news from TV, 10.6% newspaper, 7.4% social media, 24.2% online news. (all others were political/election questions)
10/18–10/20 (17 of 17)	YouGov, The Economist[28]	1500	If and when a coronavirus vaccine becomes available, will you get vaccinated?	Yes/not sure/no	42%		32%		26%	Willingness increased from last week’s 36%. 24% Black and 44% Hispanic, 36% female and 48% male; income: 37% for <50 k vs. 51% 100 k+; 48% Dem vs. 34% Rep. More likely: male, college grad, 65+, higher income, West, liberal. 40% believe available by summer 2021; 40% very and 35% somewhat concerned about safety of fast-tracked (declined from last survey).

* This table presents 70 of the 126 surveys included in the review. For space consideration, when a series has five or more surveys, only the first and last surveys of the series are presented here. A complete summary table with all 126 surveys is available from the authors. Surveys are organized and presented chronologically based on the end-date of surveys (first column). Surveys published in peer-reviewed journals as results from academic database searches are listed by first author’s last name; syndicated surveys are listed by organization (second column). ^†^ Convenience sample.
